# Cetirizine suppresses cancer-associated fibroblast-mediated fibrotic remodeling and epithelial–mesenchymal transition via histamine H_1_ receptor inverse agonism

**DOI:** 10.1186/s13058-026-02284-x

**Published:** 2026-04-26

**Authors:** Aya Sasaki, Masahiro Hosonuma, Yuki Maruyama, Eiji Funayama, Hitoshi Toyoda, Toshiaki Tsurui, Kohei Tajima, Rie Nakashima, Yoshitaka Yamazaki, Akira Orimo, Tatsunori Oguchi, Kiyoshi Yoshimura, Atsuo Kuramasu

**Affiliations:** 1 Department of Clinical Immuno Oncology, Clinical Research Institute for Clinical Pharmacology and Therapeutics, Showa Medical University, 6-11-11 Kita-karasuyama, Setagaya-ku, Tokyo, 157-8577 Japan; 2 Division of Medical Pharmacology, Department of Pharmacology, Showa Medical University School of Medicine, Tokyo, Japan; 3 Pharmacological Research Center, Showa Medical University, Tokyo, Japan; 4 Department of Surgery, Toho University Ohashi Medical Center, Tokyo, Japan; 5 Division of Medical Oncology, Department of Medicine, Showa Medical University School of Medicine, Tokyo, Japan; 6 Department of Gastroenterological Surgery, Tokai University School of Medicine, Kanagawa, Japan; 7 Department of Toxicology, Showa Medical University Graduate School of Pharmacy, Tokyo, Japan; 8 Department of Molecular Pathogenesis, Juntendo University Graduate School of Medicine, Tokyo, Japan; 9 Department of Pathology and Oncology, Juntendo University Faculty of Medicine, Tokyo, Japan; 10 Faculty of Health Sciences, Graduate School of Medicine, Juntendo University , Tokyo, Japan

**Keywords:** Triple-negative breast cancer, Epithelial–mesenchymal transition, Histamine H_1_ receptor, Cancer-associated fibroblasts, Cetirizine

## Abstract

**Background:**

Triple-negative breast cancer (TNBC) is an aggressive subtype characterized by high metastatic potential and limited therapeutic options. Cancer-associated fibroblasts (CAFs) promote epithelial–mesenchymal transition (EMT) and metastatic progression through extracellular matrix (ECM) remodeling and TGF-β signaling; however, the role of histamine H_1_ receptor (H1R) signaling in CAFs remains unclear. This study investigated whether cetirizine, a clinically approved second-generation H1R antagonist, modulates CAF function and suppresses metastasis in TNBC.

**Methods:**

Syngeneic 4T1 mouse models were used to evaluate the effects of cetirizine on tumor growth and metastasis. Bulk RNA sequencing and pathway analyses were performed on tumor tissues. Human breast cancer–derived CAFs were analyzed for H1R-dependent signaling, gene expression, and cytokine production. Non-contact co-culture assays were used to assess CAF-induced migration and EMT in 4T1 cells.

**Results:**

Cetirizine improved survival in a subcutaneous TNBC model but had no effect in a lung colonization model, indicating suppression of early metastatic processes. Transcriptomic analyses revealed downregulation of ECM remodeling and fibrotic pathways. In CAFs, cetirizine inhibited H1R-dependent calcium signaling and suppressed expression of ECM genes (COL1A1, COL3A1, FN1, HSPG2) and TGFB1. These effects persisted under histamine-depleted conditions and were abolished by HRH1 knockdown, supporting inverse agonist activity. Functionally, cetirizine inhibited CAF-induced migration and EMT marker expression in 4T1 cells without direct effects on tumor cells in monoculture.

**Conclusions:**

Cetirizine suppresses CAF-mediated fibrotic remodeling and EMT through H1R-dependent mechanisms, likely via inverse agonism. By targeting stromal components of the tumor microenvironment, cetirizine may represent a practical repurposing strategy for preventing early metastatic progression in TNBC.

**Supplementary Information:**

The online version contains supplementary material available at 10.1186/s13058-026-02284-x.

## Background

Breast cancer is the most commonly diagnosed malignancy worldwide. Among its subtypes, triple-negative breast cancer (TNBC), which lacks expression of hormone receptors and human epidermal growth factor receptor 2 (HER2), does not benefit from endocrine therapy or HER2-targeted therapy, leaving chemotherapy as the mainstay of treatment. TNBC is more frequently diagnosed in younger patients, exhibits a high propensity for recurrence and metastasis, and is associated with poor prognosis [8]. Immune checkpoint inhibitors (ICIs) have been introduced into the treatment of TNBC and have demonstrated clinical benefits in selected patients [1]. However, the overall response rates remain limited, underscoring the urgent need to develop more effective combination strategies [24]. In addition, advances in precision medicine have revealed substantial heterogeneity in TNBC and the contribution of genetic and molecular determinants to therapeutic resistance [23].

The tumor microenvironment (TME) plays a pivotal role in regulating tumor progression through stromal remodeling. In particular, cancer-associated fibroblasts (CAFs) are key regulators of the tumor stroma and drive extracellular matrix (ECM) remodeling and fibrotic processes through the production of transforming growth factor-β (TGF-β), secretion of diverse cytokines, and deposition of ECM components [3, 15]. These CAF-driven fibrotic changes are now recognized as major determinants of tumor progression and therapeutic resistance, and promote epithelial–mesenchymal transition (EMT), a process closely associated with the high metastatic potential of TNBC [19]. EMT confers invasive and migratory capabilities and is associated with stem cell–like properties and resistance to both chemotherapy and immune checkpoint inhibitors [9]. In addition, CAFs contribute to the establishment of an immunosuppressive milieu that hampers T-cell infiltration and limits the efficacy of ICI therapy. Thus, CAF-mediated stromal remodeling represents a critical therapeutic target linking metastasis and immune evasion.

Histamine, a well-established mediator of inflammation and allergic responses, also regulates fibroblast function [18]. In particular, histamine H_1_ receptor (H1R, gene symbol: HRH1) signaling promotes fibroblast activation and fibrosis, thereby contributing to wound healing and tissue remodeling [25]. Although several studies have reported antitumor effects of H1R antagonists [5, 12], including suppression of tumor cell migration and EMT [4, 21], these effects have largely been attributed to tumor cell–intrinsic mechanisms. In contrast, the role of H1R signaling in stromal cells, particularly in CAF-mediated regulation of fibrotic remodeling and its downstream impact on EMT, remains poorly understood.

In this study, we focused on cetirizine, a widely used second-generation H1R antagonist, to investigate its potential antimetastatic effects in TNBC models and to elucidate the underlying molecular mechanisms. Specifically, we aimed to clarify the role of H1R signaling in CAF-mediated regulation of fibrotic remodeling and its downstream impact on EMT, and to explore the potential of cetirizine to enhance antitumor efficacy when combined with immune checkpoint blockade.

## Methods

### TCGA data analysis

Transcriptomic and clinical data from The Cancer Genome Atlas (TCGA) Breast Invasive Carcinoma (BRCA) cohort were analyzed using the UCSC Xena browser (https://xena.ucsc.edu). Samples with available data for estrogen receptor (ER) status (ER_status_nature2021), progesterone receptor (PR) status (PR_status_nature2021), HER2 final status (HER2_final_status_nature2021), and HRH1 expression were initially selected (n = 744). From these, samples with indeterminate ER or PR status and those with equivocal HER2 status were excluded, leaving 728 samples for subtype classification. Breast cancer subtypes were defined as follows: Luminal subtype included samples that were positive for either ER or PR (n = 575); HER2-enriched subtype included samples that were HER2-positive and ER-/PR-negative (n = 30); and TNBC included samples that were negative for ER, PR, and HER2 (n = 123). For Kaplan–Meier survival analyses, samples with overall survival time equal to zero (OS.time = 0) were excluded. After this additional filtering, 562 Luminal cases were included in the survival analysis. For each subtype, samples were stratified into HRH1-high and HRH1-low groups based on the top and bottom 50% of HRH1 expression levels, respectively. Kaplan–Meier survival analysis was performed to compare overall survival between these two groups within each subtype. Gene set enrichment analysis (GSEA) was conducted using blitzGSEA [11] to identify Hallmark pathways enriched in the HRH1-high group for each subtype. In addition, for the TNBC cohort, expression data for the 195 genes in the HALLMARK EPITHELIAL MESENCHYMAL TRANSITION gene set (MSigDB) were obtained via UCSC Xena. To evaluate the association between HRH1 and EMT-related gene expression, we calculated Spearman’s rank correlation coefficients (ρ) and corresponding *P*-values between HRH1 and each EMT gene using the scipy.stats.spearmanr function in Python. Genes with ρ > 0.4 were considered to show moderate to strong positive correlation with HRH1.

### Mouse subcutaneous tumor model

BALB/c mice (female, 8–12 weeks old, average body weight ~ 22 g) were purchased from CLEA Japan (Tokyo, Japan) and maintained at a constant temperature (23 ± 1 °C) with a 12 h light/dark cycle under specific pathogen-free conditions. The experimental protocols were approved by the Institutional Animal Care and Use Committee of Showa Medical University, and all experiments were conducted in accordance with institutional guidelines and regulations. 4T1 cells were cultured in RPMI1640 medium supplemented with 10% fetal bovine serum (FBS; Thermo Fisher Scientific, Waltham, MA, USA) and 1% penicillin–streptomycin (Thermo Fisher Scientific) at 37 °C in a humidified atmosphere containing 5% CO_2_. Cells were passaged every 2–3 days to maintain exponential growth and were confirmed to be mycoplasma-free prior to use. For tumor inoculation, 4T1 cells (1 × 10^5^ cells in 50 μL Hanks’ Balanced Salt Solution [HBSS]) were injected subcutaneously into the right flank, as previously described [22]. Cetirizine (LKT Labs, St. Paul, MN, USA) was administered via drinking water at a concentration of 0.1 g/L, corresponding to an approximate dose of 20 mg/kg/day based on average water intake and body weight [14]. In experiments testing the effect of cetirizine alone, administration was initiated either 7 days prior to tumor inoculation (prophylactic regimen) or on the day of tumor inoculation (therapeutic regimen) and continued until the end of the experiment. In combination therapy experiments, cetirizine administration began 14 days prior to tumor inoculation and continued throughout the study. Mice had free access to cetirizine-containing water, and there was no significant difference in water consumption between the cetirizine-treated and control groups. For immune checkpoint blockade, mice received intraperitoneal injections of anti-PD-1 antibody (clone RMP1-14, BioXcell, Lebanon, NH) or control rat IgG (FUJIFILM Wako, Osaka, Japan) at a dose of 150 μg per mouse, administered once a week starting on day 7. Tumor length and width were measured twice weekly, and tumor volume was calculated as (length × width^2^ × 0.5). Mice were monitored daily for general condition and survival until day 28 after tumor inoculation. Spontaneous deaths were recorded during the observation period. For tumor growth analyses, only mice that survived until day 17 after tumor inoculation were included in the calculation of mean tumor volumes; data from mice that died before day 17 were excluded from tumor size comparisons. On day 28 following tumor inoculation, mice were euthanized for tissue collection.

### Lung metastasis model

A lung metastasis model was established by intravenous injection of 4T1 cells via the tail vein. Female BALB/c mice (8–12 weeks old) were injected with either 1 × 10^4^ or 1 × 10^5^ 4T1 cells suspended in 100 μL phosphate-buffered saline. Cetirizine and anti-PD-1 antibody were administered following the same dosing schedules as described for the subcutaneous tumor model. Mice were monitored daily for signs of distress and euthanized at a predefined endpoint or when humane criteria were met.

### RNA sequencing

The 4T1 subcutaneous tumor model was established as described above. On day 14 after tumor implantation, tumors were excised and total RNA was extracted using the RNeasy Mini Kit (Qiagen, Hilden, Germany) according to the manufacturer’s instructions. Bulk RNA sequencing (RNA-seq) was outsourced to Rhelixa, Inc. (Tokyo, Japan). Libraries were prepared using poly(A) selection, and sequencing was performed on an Illumina NovaSeq 6000 platform. The resulting transcriptomic data were analyzed using Ingenuity Pathway Analysis (IPA, Qiagen) to identify enriched canonical pathways.

### Flow cytometric analysis of tumor microenvironment

Subcutaneous 4T1 tumors were established as described above. On day 14 after inoculation, tumors were harvested and immediately processed for flow cytometric analysis [7]. Excised tumors were minced into small pieces and digested in RPMI-1640 containing 0.02 mg/mL DNase I and 0.1 mg/mL Liberase TM (Roche, Mannheim, Germany) at 37 °C for 1 h with agitation. The digested samples were then passed through a cell strainer to obtain single-cell suspensions and remove debris. For analysis of overall TME cellular composition, whole tumor cell suspensions were directly stained with antibody panel 1. For immune cell analysis, mononuclear cells were enriched using a density gradient. Briefly, cell suspensions were layered onto a Ficoll-Paque PLUS (Cytiva, Marlborough, MA, USA; Cat. No. 17-1440-02) and centrifuged at 400 ×g for 20 min at room temperature. Cells were recovered from the interface and stained with antibody panel 2. Panel 2 consisted of two subpanels: (2–1) antigen-presenting cells and (2–2) T cells. Detailed lists of antibodies used in each panel are provided in Supplementary Material 5. Gating strategies for TME cellular composition and tumor-infiltrating immune cells are shown in Supplementary Figures S3 and S4 (Supplementary Material 6), respectively.

### Single cell RNA-seq data analysis

Single-cell RNA sequencing data from human breast cancer samples [26] were accessed and visualized through the Single Cell Portal (https://singlecell.broadinstitute.org/single_cell). Cells were categorized by the “Cell type major” annotation provided in the dataset. Gene expression of selected target genes was visualized using dot plots, where dot size represents the percentage of cells expressing the gene and color intensity reflects average expression level.

### Cell culture and H1R antagonist treatment

CAFs originally established from human breast tumor tissue as previously described [10] were maintained in DMEM (Fujifilm Wako) supplemented with 10% FBS and 1 × penicillin–streptomycin at 37 °C in a humidified atmosphere containing 5% CO_2_. Human umbilical vein endothelial cells (HUVECs; Lifeline Cell Technology, San Diego, CA, USA) were cultured in HuMedia-EB2 medium supplemented with 2% FBS, 10 ng/mL human epidermal growth factor, 1.34 μg/mL hydrocortisone hemisuccinate, 50 μg/mL gentamicin, 50 ng/mL amphotericin B, 5 ng/mL human basic fibroblast growth factor, and 10 μg/mL heparin, at 37 °C in 5% CO_2_. MDA-MB-231 human breast cancer cells were maintained in DMEM supplemented with 10% FBS and 1 × penicillin–streptomycin at 37 °C in 5% CO_2_. Cells were seeded in 6-well plates at a density of 2 × 10^6^ cells per well. On the following day, cetirizine or fexofenadine (TCI, Tokyo, Jpanan; Cat. No. F0698) were added to the culture medium at the indicated concentrations. After 4 h of incubation, total RNA was extracted using the RNeasy Mini Kit (Qiagen) according to the manufacturer’s instructions. For CAFs, culture supernatants were additionally collected after 24 h of treatment for measurement of TGFβ1. Latent TGFβ1 was activated by acidification with HCl, and total TGFβ1 concentrations were quantified by enzyme-linked immunosorbent assay (ELISA) using Human TGF-β1 DuoSet Kit (DY240, R&D Systems).

### Intracellular Ca^2+^ measurement

CAFs were seeded in clear-bottom black-wall 96-well plates one day prior to the assay. On the day of measurement, cells were washed with assay buffer consisting of 1 × HBSS (with Ca^2+^) supplemented with 0.1% bovine serum albumin (BSA) and 2.5 mM probenecid. Cells were incubated with Fluo-3 AM (2 µM; Dojindo Laboratories, Kumamoto, Japan; Cat. No. F026) and Pluronic F-127 (0.04%; supplied with the Fluo-3 AM reagent) in assay buffer for 30 min at 37 °C. After dye loading, cells were washed twice with assay buffer. Intracellular Ca^2+^ dynamics were measured using a FlexStation 3 (Molecular Devices) at 37 °C. Histamine (TCI, H0146) and cetirizine were applied at the indicated concentrations, and fluorescence signals were recorded over time. Relative changes in intracellular Ca^2+^ levels were expressed as Δ(F − F₀)/F₀, where F₀ represents the mean fluorescence intensity before stimulation and F represents the fluorescence intensity at each time point.

### siRNA-mediated knockdown

Small interfering RNA (siRNA) targeting human HRH1 (sc-35563) was purchased from Santa Cruz Biotechnology (Dallas, TX, USA). A non-targeting control siRNA (sc-37007; Santa Cruz Biotechnology) was used as a negative control. Cells were transfected with siRNA using ScreenFect siRNA (Fujifilm Wako) according to the manufacturer’s instructions.

### Histamine depletion conditions

For histamine depletion, CAFs were seeded in 6-well plates and cultured in complete medium. Cells were treated with α-fluoromethylhistidine (α-FMH; 100 μM), an irreversible inhibitor of histidine decarboxylase, for ≥ 24 h to suppress endogenous histamine synthesis [2]. α-FMH was kindly provided by Prof. Kazutaka Maeyama (Ehime University, Japan). Because FBS may contain histamine, cells were washed twice and subsequently maintained in serum-free RPMI containing 0.1% BSA and α-FMH (100 μM) to minimize exogenous histamine. Cetirizine (1 μM) was then added, and total RNA was harvested 4 h later.

### Quantitative reverse transcription-polymerase chain reaction (RT-PCR)

For each sample, 250 ng of total RNA was reverse-transcribed using the High-Capacity cDNA Reverse Transcription Kit (Thermo Fisher Scientific) with random hexamers, following the manufacturer’s protocol. Multiplex quantitative PCR was performed on a QuantStudio 3 Real-Time PCR System using TaqMan Fast Advanced Master Mix (Thermo Fisher Scientific), as previously described [17]. Predesigned PrimeTime qPCR assays (FAM-labeled) for TGFB1(Hs.PT.58.39813975), COL1A1 (Hs.PT.58.15517795), COL3A1 (Hs.PT.58.4249241), FN1 (Hs.PT.58.40005963), HSPG2 (Hs.PT.58.18698732), HRH1 (Hs.PT.58.39265204), Vim (Mm.PT.58.8720419), and Acta2 (Mm.PT.58.16320644) were purchased from Integrated DNA Technologies (Coralville, IA, USA). For mouse samples, Actb (β-actin, VIC-labeled) was used as the endogenous control, while for human samples, HPRT (HEX-labeled) was used. Relative gene expression was calculated using the comparative Ct (ΔΔCt) method and expressed as fold change relative to the control sample.

### Transwell co-culture assay

Transwell co-culture experiments were performed using 24-well plates. CAFs were seeded in the lower chamber at a density of 3 × 10^5^ cells per well in DMEM containing 1% FBS. A transwell insert (Chemotaxicell, pore size: 8 µm; Kurabo, Osaka, Japan) was placed in each well, and 4T1 cells were seeded into the upper chamber at a density of 3 × 10^5^ cells per insert in DMEM without serum. Cetirizine was added at a final concentration of 1 µM. After 24 h of co-culture, 4T1 cells were collected from the upper chamber, and total RNA was extracted using the RNeasy Mini Kit (Qiagen) according to the manufacturer’s instructions. For the migration assay, transwell co-cultures were maintained for 72 h. Non-migrated cells on the upper surface of the Chemotaxicell membrane were removed, and the membrane was fixed and stained with 1% crystal violet (TCI; Cat. No. C0428). Migrated cells on the lower surface were visualized using a Keyence microscope (BZ-X800), and migration was quantified by measuring the stained cell area with the Hybrid Cell Count analysis software (BZ-H4A and BZ-HRH14C; Keyence, Osaka, Japan).

### Flow cytometric analysis of vimentin expression

CAFs were transfected with HRH1-specific siRNA or control siRNA. After 24 h, the culture medium was replaced, and cetirizine (1 μM) was added as indicated. Cells were subsequently co-cultured with 4T1 cells using a non-contact transwell system for 6 h prior to analysis. Cells were stained for intracellular vimentin using the BD Transcription Factor Buffer Set (BD Biosciences, San Jose, CA, USA; Cat. No. 562574) according to the manufacturer’s instructions. Briefly, cells were fixed, permeabilized, and incubated with purified anti-vimentin antibody (rat monoclonal, clone W16220A; BioLegend, San Diego, CA, USA), followed by FITC-conjugated goat anti-rat IgG secondary antibody (minimal cross-reactivity; BioLegend). IgG from rat serum (I4131; Sigma-Aldrich, St. Louis, MO, USA) was used as an isotype control. After staining, cells were washed and analyzed by flow cytometry. Data were expressed as geometric mean fluorescence intensity (GeoMFI).

### Statistical analysis

Tumor growth was compared between groups using a mixed-effects analysis followed by Tukey’s multiple comparison test. Survival curves were compared using the log-rank test. For comparisons among three or more groups, repeated-measures one-way analysis of variance (ANOVA) with Dunnett’s or Bonferroni’s multiple comparison test, or Kruskal–Wallis test with Dunn’s multiple comparison test, was used as appropriate depending on experimental design. Data are presented as mean ± standard erroe of the mean (SEM). Statistical analyses were performed using GraphPad Prism 10 for Mac OS (GraphPad Software, San Diego, CA, USA). A *p* value of < 0.05 was considered statistically significant. Correlation analyses between tumor volume and the HRH1-related EMT gene score were performed using Spearman’s rank correlation coefficient with exact two-tailed *P* values.

## Results

### HRH1 expression predicts poor prognosis in TNBC and is linked to EMT and immune-related pathways

**Fig. 1:**
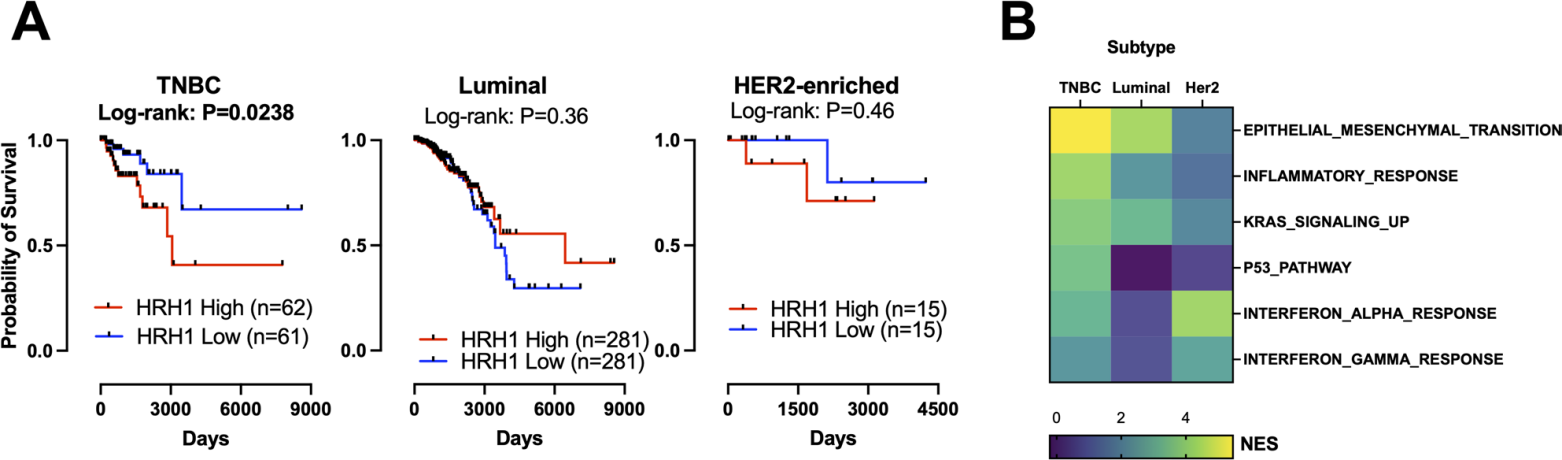
HRH1 expression predicts poor prognosis in TNBC. **A** Kaplan–Meier survival curves of overall survival in TNBC (left), Luminal (middle), and HER2-enriched (right) subtypes from the TCGA BRCA cohort. Patients were stratified into HRH1-high (red) and HRH1-low (blue) groups. **B** Heatmap of Hallmark pathways enriched in the HRH1-high group (NES > 2) in each subtype based on gene set enrichment analysis (GSEA). TNBC, triple-negative breast cancer; NES, normalized enrichment score

We first examined whether HRH1 expression was associated with prognosis in different breast cancer subtypes. Transcriptomic and clinical data from the TCGA Breast Invasive Carcinoma (BRCA) cohort (n = 744) were analyzed. Patients were categorized into TNBC (n = 123), Luminal (n = 562), and HER2-enriched (n = 30) subtypes. Kaplan–Meier analysis showed that in TNBC, low HRH1 expression was associated with significantly better overall survival, whereas no significant survival difference was observed between HRH1-high and HRH1-low groups in the Luminal or HER2-enriched subtypes (Fig. 1A).

To explore the molecular basis underlying this subtype-specific effect, we next performed gene set enrichment analysis (GSEA). In TNBC, five Hallmark pathways were strongly enriched in the HRH1-high group with a normalized enrichment score (NES) greater than 2: Epithelial Mesenchymal Transition, Inflammatory Response, KRAS Signaling Up, P53 Pathway, and Interferon Alpha Response. Comparison of NES values across the three subtypes (Fig. 1B) highlighted that EMT-related signatures were most prominently enriched in TNBC. In addition, the enrichment of inflammatory and interferon-related pathways suggested that HRH1 signaling may influence both tumor progression and tumor–immune interactions. A full list of GSEA results stratified by the three breast cancer subtypes is presented in Supplementary Material 1.

### Cetirizine monotherapy improves survival in a syngeneic TNBC mouse model

**Fig. 2:**
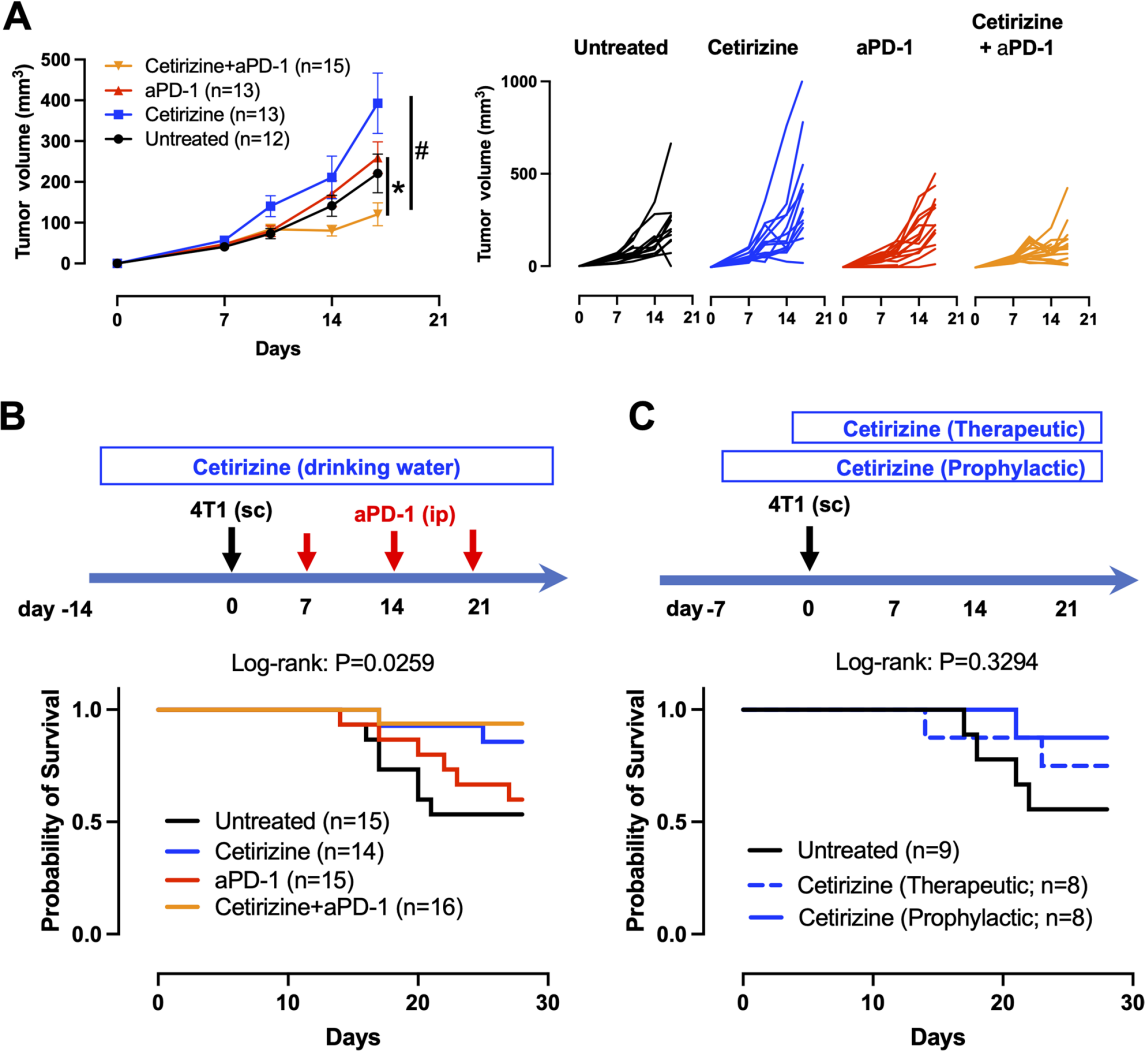
Cetirizine monotherapy improves survival in the 4T1 TNBC mouse model. **A** Tumor growth curves of subcutaneously implanted 4T1 cells comparing untreated, cetirizine monotherapy, aPD-1 monotherapy, and cetirizine + aPD-1 combination therapy groups. Left: mean ± SEM. Right: individual tumor growth curves for each mouse. #*p* < 0.05 Combination vs. Cetirizine alone; *p < 0.05 Combination vs. anti-PD-1 alone. **B** Kaplan–Meier survival curves of the same four groups as in (**A**). The experimental schedule is shown above. **C** Kaplan–Meier survival curves comparing survival outcomes according to the timing of cetirizine administration. The experimental schedule is shown above. aPD-1, anti-programmed cell death-1 antibody; sc, subcutaneous; ip, intraperitoneal. The number of mice in each group is indicated in the graph legend. For tumor growth analyses, mice that died before day 17 were excluded from tumor size calculations, resulting in differences in sample size between tumor growth and survival analyses

Based on these results, we hypothesized that suppression of H1R activity might exert inhibitory effects on TNBC progression and potentially ameliorate the immunosuppressive tumor microenvironment that limits the efficacy of immune checkpoint inhibitors (ICIs). To test this hypothesis, we evaluated the therapeutic effects of cetirizine, a second-generation H1R antagonist, in combination with anti-PD-1 (aPD-1) antibody in a syngeneic TNBC mouse model using 4T1 cells (Fig. 2). In the subcutaneous tumor model, aPD-1 monotherapy had no effect on tumor growth. However, the addition of cetirizine to aPD-1 suppressed tumor growth compared with aPD-1 alone. Cetirizine monotherapy showed a tendency toward enhanced tumor growth, although the difference was not statistically significant (Fig. 2A). During the course of the experiment, spontaneous deaths were observed in a subset of mice across all treatment groups. At necropsy, hemorrhagic pleural effusion was consistently detected in all deceased animals. Although macroscopically visible metastatic lesions were not observed, histological examination of excised lung tissues revealed the presence of tumor cells in a subset of cases (Supplementary Figure S1, Supplementary Material 6). These findings suggest that systemic disease progression, including pulmonary involvement, may have contributed to mortality in this model; however, the precise cause of death could not be definitively determined.

Survival analysis revealed that cetirizine monotherapy significantly improved survival compared with the untreated group (Fig. 2B). Although the combination therapy also improved survival relative to controls, the effect was only marginally better than that of cetirizine monotherapy. Notably, mice treated with cetirizine alone exhibited better survival outcomes than those treated with aPD-1 alone. In a separate experiment, when cetirizine administration was initiated immediately after tumor implantation, survival tended to be prolonged compared with controls, although the difference did not reach statistical significance (Fig. 2C). Together, these results suggest that while combination therapy provides the greatest control of local tumor growth, cetirizine monotherapy alone is sufficient to confer a significant survival benefit in this TNBC model.

**Fig. 3:**
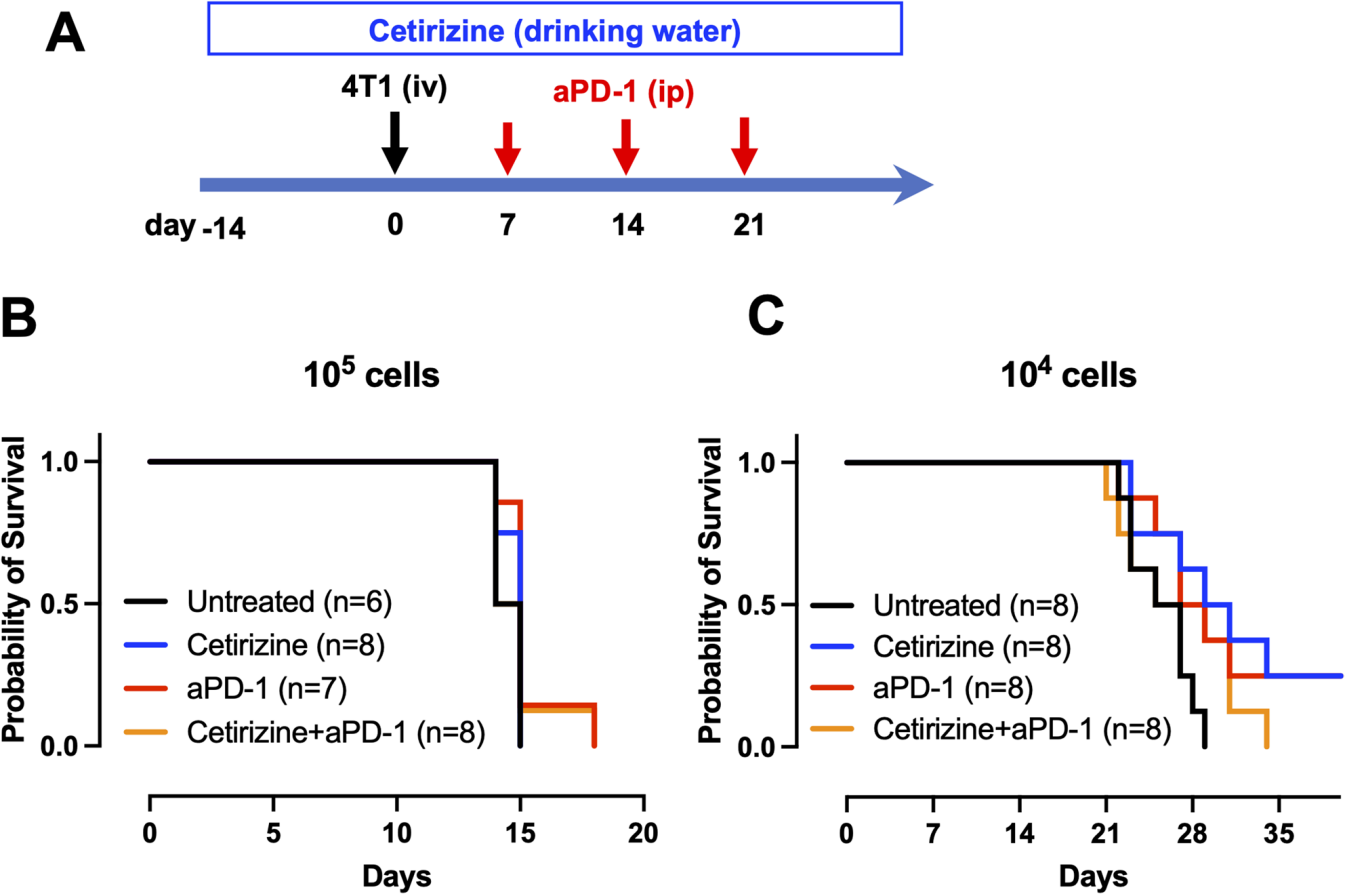
Cetirizine has no therapeutic effect in the lung metastasis model. **A** Experimental schedule of the lung metastasis model. **B**, **C** Kaplan–Meier survival curves comparing untreated, cetirizine monotherapy, aPD-1 monotherapy, and cetirizine + aPD-1 combination therapy groups. Mice were injected with 1 × 10^5^ (**B**) or 1 × 10^4^ (**C**) 4T1 cells via the tail vein. aPD-1, anti-programmed cell death-1 antibody; iv, intravenous; ip, intraperitoneal. The number of mice in each group is indicated in the graph legend

To better understand which stage of the metastatic process was affected by cetirizine, we next employed an experimental lung metastasis model in which 4T1 cells were injected into the tail vein to directly establish pulmonary metastases, thereby bypassing primary tumor growth and early dissemination steps and focusing on later stages such as extravasation and colonization (Fig. 3A, Supplementary Figure S2, Supplementary Material 6). In this model, neither cetirizine monotherapy nor combination therapy with aPD-1 improved overall survival (Fig. 3B). This lack of efficacy was consistent even when the number of injected tumor cells was reduced, indicating that cetirizine did not improve survival in this model, suggesting a limited effect on later metastatic steps such as colonization (Fig. 3C). These findings indicate that the beneficial effects of cetirizine observed in the subcutaneous model are unlikely to be primarily mediated by inhibition of later metastatic steps such as extravasation or colonization. Instead, cetirizine may preferentially interfere with earlier stages of the metastatic cascade, potentially including processes associated with EMT.

### Cetirizine suppresses EMT-related programs in tumors

To further elucidate the mechanisms by which cetirizine suppresses metastasis and to gain insight into its potential role in local tumor control when combined with aPD-1, we performed bulk RNA-seq of tumors harvested 14 days after subcutaneous implantation. The complete gene expression profiles for all four groups are provided in Supplementary Material 2.

**Fig. 4:**
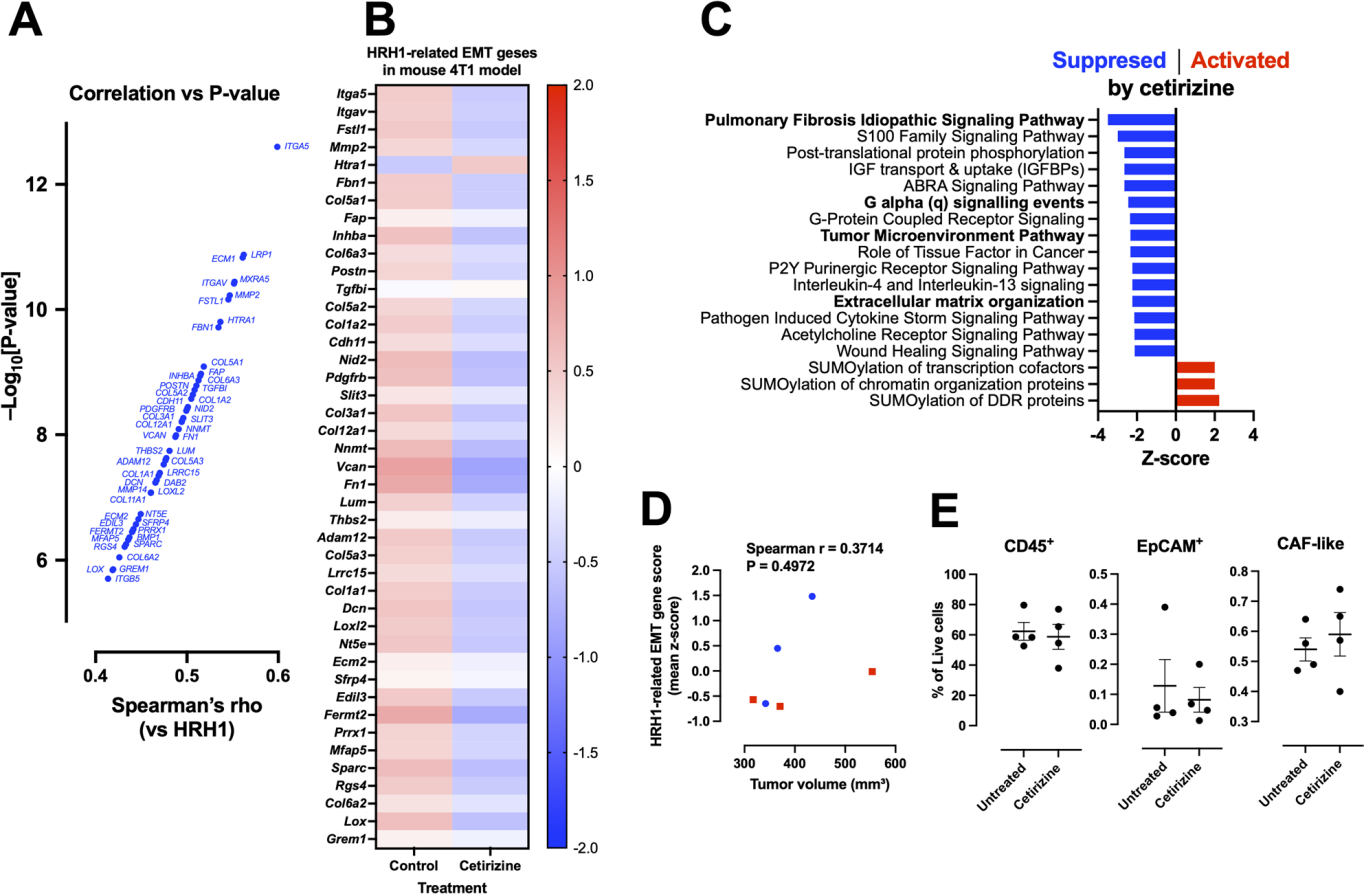
Downregulation of EMT-related pathways in cetirizine-treated tumors. **A** Correlation between HRH1 expression and EMT-related genes (Hallmark EMT signature, 195 genes) in the TCGA TNBC cohort. The x-axis shows Spearman’s ρ and the y-axis shows the correlation P-value. Only genes with ρ > 0.4 are displayed, with gene names labeled. The full list is provided in Supplementary Material 3. **B** Heatmap of the mouse orthologs of the 51 EMT-related genes (ρ > 0.4 in A) based on bulk RNA-seq data from the 4T1 subcutaneous tumor model, comparing untreated (Control) and cetirizine-treated groups. **C** Ingenuity Pathway Analysis (IPA) of bulk RNA-seq data comparing cetirizine-treated and untreated tumors. Canonical pathways with activation z-scores > 2 (red) or < –2 (blue) are shown. Positive z-scores indicate activation in the cetirizine-treated group, whereas negative z-scores indicate activation in the untreated group. Pathways discussed in the main text are shown in bold. **D** Correlation between tumor volume and EMT score in the 4T1 model (untreated and cetirizine-treated tumors). Each point represents an individual tumor; blue and red symbols denote untreated and cetirizine-treated tumors, respectively. Spearman’s correlation coefficient and corresponding P-value are shown in the plot. **E** Flow cytometric analysis of tumor cellular composition, showing proportions of immune cells (CD45^+^), tumor-enriched cells (EpCAM^+^), and CAF-like cells in 4T1 tumors

We first assessed the relationship between HRH1 expression and EMT-related genes in the TCGA TNBC cohort. Among the 195 genes comprising the Hallmark EMT signature, 131 showed a positive correlation with HRH1 expression (Spearman ρ > 0.2) and 3 showed a negative correlation (ρ < –0.2) (full list in Supplementary Material 3). Figure 4A highlights the 51 genes with a moderate positive correlation (ρ > 0.4) with HRH1 expression. We then examined the expression of the corresponding mouse orthologs of these genes in our 4T1 tumor model. A majority of these EMT-associated genes were downregulated in tumors treated with cetirizine compared with untreated controls (Fig. 4B).

Furthermore, Ingenuity Pathway Analysis (IPA) confirmed that pathways related to extracellular matrix remodeling and the tumor microenvironment were significantly suppressed in the cetirizine-treated group (Fig. 4C; full list in Supplementary Material 4), including the Tumor Microenvironment Pathway (activation z-score = –2.333) and Extracellular Matrix Organization (–2.236). Strong suppression of the Pulmonary Fibrosis Idiopathic Signaling Pathway (–3.5) was also observed, consistent with an antifibrotic effect. These findings suggest that cetirizine modulates fibrotic and microenvironmental programs within tumors, which may indirectly contribute to the suppression of EMT through modulation of the tumor microenvironment. In addition, the G alpha (q) Signaling Events pathway (–2.449), which involves signaling downstream of the H1R, was downregulated in the cetirizine-treated tumors, indicating inhibition of H1R–mediated signaling.

EMT gene scores did not correlate with tumor volume across individual tumors (Fig. 4D), indicating that EMT suppression was not simply a consequence of differences in tumor size. Furthermore, flow cytometric analysis of tumors showed that the proportions of tumor cells, CAF-like stromal cells, and CD45^+^ immune cells were comparable among treatment groups (Fig. 4E), with no significant differences in the relative abundance of these major cellular compartments. Collectively, these findings suggest that the observed changes are unlikely to be explained by differences in bulk cellular composition, immune infiltration, or tumor size, and are instead consistent with transcriptional modulation within the tumor microenvironment, particularly in stromal compartments.

### Combination therapy enhances metabolic and immune pathways

**Fig. 5:**
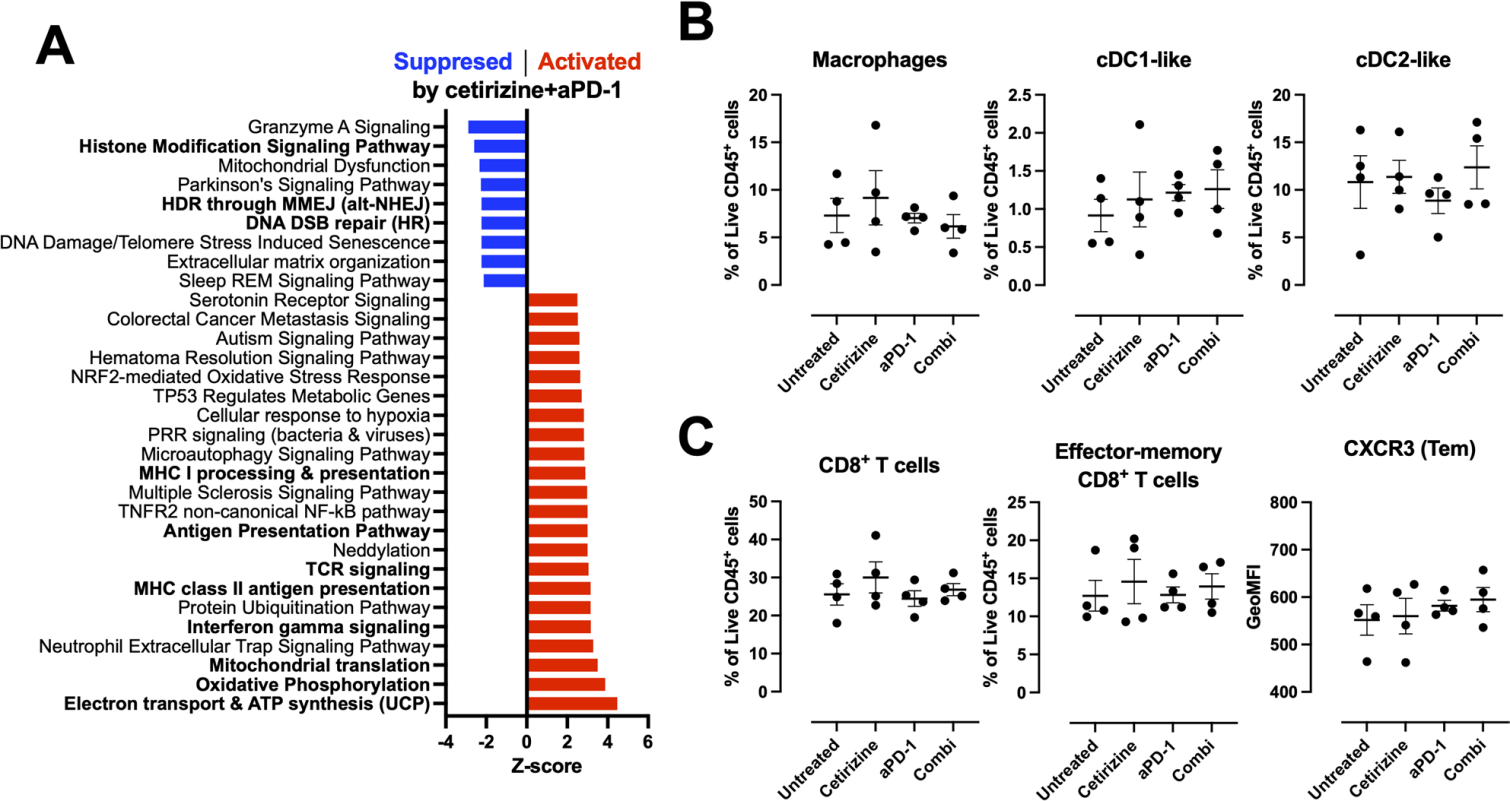
Immune-related changes in the tumor microenvironment following combination therapy. **A** Ingenuity Pathway Analysis (IPA) of bulk RNA-seq data comparing cetirizine + aPD-1–treated tumors with untreated controls. Canonical pathways with activation z-scores > 2 (red) or < –2 (blue) are shown. Positive z-scores indicate activation in the combination-treated group, whereas negative z-scores indicate activation in the untreated group. Pathways discussed in the main text are shown in bold. **B**–**C** Flow cytometric analysis of tumor-infiltrating immune cell populations in the 4T1 tumor model. The proportions of macrophages (left in B), cDC1-like cells (middle in **B**), and cDC2-like cells (right in **B**), as well as CD8^+^ T cells (left in **C**) and effector memory CD8^+^ T cells (middle in **C**), and CXCR3 geometric mean fluorescence intensity (GeoMFI) on effector memory CD8^+^ T cells (right in **C**) are shown. Combi, cetirizine + anti-PD-1 antibody; Tem, effector memory CD8^+^ T cells.

To explore the mechanism of local tumor suppression by cetirizine plus aPD-1 therapy (Fig. 2A), pathway analysis identified significantly altered canonical pathways in the combination group. Activated pathways were primarily associated with two axes: metabolic normalization and immune activation (Fig. 5A). The metabolic axis included electron transport, ATP synthesis, oxidative phosphorylation, and mitochondrial translation, consistent with enhanced mitochondrial function. The immune axis included antigen presentation (MHC class I and II), TCR signaling, and interferon-γ signaling, indicating activation of T cell–mediated immunity. In contrast, suppressed pathways were mainly related to DNA repair, including DNA damage–induced senescence, homologous recombination, alternative end joining, and histone modification. Together, these results suggest that cetirizine plus aPD-1 therapy may contribute to tumor control by enhancing mitochondrial and immune-related pathways while impairing the DNA repair capacity of tumor cells.

To directly assess whether these immune-related transcriptomic changes reflected alterations in tumor-infiltrating immune cell composition, we performed flow cytometric analysis of tumors on day 14, corresponding to the time point used for bulk RNA-seq (Fig. 5B, C). The proportions of intratumoral dendritic cell subsets (cDC1 and cDC2), macrophages, and CD8^+^ T cell memory fractions (CD44^+^/CD62L^–^) were not significantly different among groups. Furthermore, CXCR3 expression levels on effector memory CD8^+^ T cells were comparable between treatment groups. These findings indicate that the immune pathway enrichment observed in bulk RNA-seq does not reflect marked quantitative changes in immune cell infiltration at this time point. Rather, the data suggest functional modulation within existing tumor-infiltrating immune cells and/or tumor cell–intrinsic upregulation of antigen presentation pathways.

### H1R–mediated suppression of EMT-related gene expression in breast cancer-associated fibroblasts

**Fig. 6:**
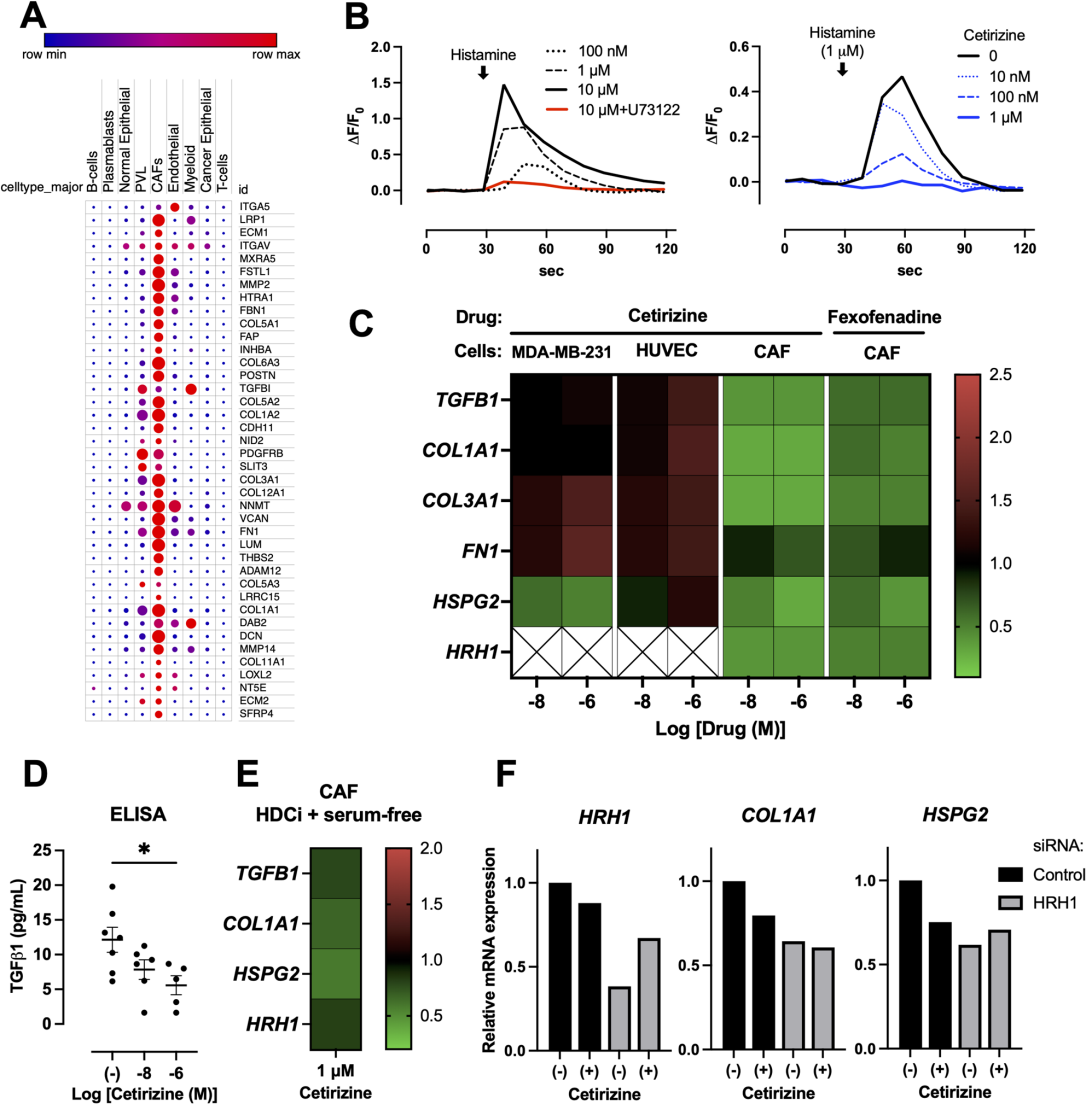
Cetirizine suppresses EMT-related gene expression in breast cancer–associated fibroblasts. **A** Dot plot based on human breast cancer single-cell RNA-seq data (Wu et al.), showing the expression patterns of the top 40 genes most strongly correlated with HRH1 in the TCGA TNBC cohort. Major cell types are shown at the top, and gene IDs are listed on the right, ordered from top (highest correlation, rank 1) to bottom (rank 40). Dot size indicates the proportion of cells expressing each gene, and dot color reflects the average expression level. CAFs, cancer-associated fibroblasts; PVL, perivascular-like cells. **B** Cetirizine inhibits histamine-induced calcium responses in CAFs. (Left) Effect of histamine concentration (100 nM–10 μM) on intracellular Ca^2+^ responses (ΔF/F₀). The red trace represents the response to 10 μM histamine in the presence of U73122 (10 μM), a phospholipase C inhibitor. (Right) Effect of cetirizine (10 nM–1 μM) on histamine (1 μM)–induced Ca^2+^ responses. **C** Heatmap showing the effects of two H1R antagonists, cetirizine and fexofenadine, on EMT-related and HRH1 gene expression in human breast cancer cells (MDA-MB-231), endothelial cells (HUVEC), and CAFs. Relative expression levels are presented as fold changes compared with the no-antagonist condition. Numbers shown below indicate antagonist concentrations (–8 = 10 nM, –6 = 1 μM). Cells marked with “X” indicate conditions that were not measured. **D** Concentration of TGFβ1 in CAF culture supernatants measured by ELISA. Each dot represents an individual sample; bars indicate mean ± SEM. *P < 0.05 vs. untreated, as determined by Kruskal–Wallis test followed by Dunn’s multiple comparison test. **E** Heatmap showing changes in gene expression in CAFs treated with cetirizine (1 μM) under histidine decarboxylase inhibition (HDCi; α-fluoromethylhistidine, 100 μM) and serum-free conditions. Relative expression levels are presented as fold changes compared with the no-antagonist condition. **F** Quantitative RT-PCR analysis of HRH1, COL1A1, and HSPG2 expression in CAFs. The effect of cetirizine (1 μM) was evaluated under HRH1 knockdown conditions using siRNA.

To identify the cellular targets of cetirizine in the TME, we first analyzed publicly available single-cell RNA-seq data of breast cancer [26], focusing on the top 40 genes correlated with HRH1 expression in the TCGA TNBC cohort. Many of these genes were predominantly expressed in CAFs (Fig. 6A). Guided by this observation, we examined the direct effects of cetirizine on human breast cancer–derived fibroblasts [10].

To determine whether CAFs express functional H1R, we assessed intracellular calcium responses as a readout of canonical Gq signaling (Fig. 6B). Histamine induced a transient, concentration-dependent increase in intracellular Ca^2+^ levels in CAFs. This response was completely abolished by the phospholipase C inhibitor U73122. Moreover, histamine-induced Ca^2+^ responses were suppressed by cetirizine in a concentration-dependent manner. These findings indicate that CAFs express functional H1R.

Quantitative RT-PCR revealed that cetirizine suppressed the expression of extracellular matrix genes (COL1A1, COL3A1, FN1, and HSPG2) and the EMT-inducing factor TGFB1 in a concentration-dependent manner (Fig. 6C). Consistently, ELISA demonstrated a dose-dependent reduction of secreted TGFβ1 in culture supernatants (Fig. 6D). Cetirizine also reduced HRH1 expression in CAFs (Fig. 6C). Consistent with previous reports that HRH1 expression is positively regulated by its own signaling activity [16], this finding supports the notion that cetirizine suppresses basal H1R signaling.

To rule out a potential contribution of endogenous histamine, CAFs were treated under histamine-depleted conditions using the histidine decarboxylase inhibitor αFMH in combination with serum-free culture. Under these conditions, cetirizine still suppressed the expression of TGFB1, extracellular matrix, and HRH1 genes, suggesting that its effects are mediated through inverse agonism of the H1R (Fig. 6E). Furthermore, knockdown of HRH1 by siRNA reduced the expression of HRH1, COL1A1, and HSPG2, indicating that the expression of these genes depends on H1R activity. In HRH1-knockdown cells, cetirizine no longer exerted additional suppressive effects, supporting that H1R is the primary target of cetirizine in CAFs (Fig. 6F).

Similar reductions in TGFB1, COL1A1, COL3A1, HSPG2, and HRH1 were observed with fexofenadine, another H1R antagonist (Fig. 6C), supporting an H1R–dependent mechanism. In contrast, cetirizine had little or no suppressive effect on EMT-related genes in TNBC cells (MDA-MB-231) and endothelial cells (HUVECs), except for a modest reduction of HSPG2 in MDA-MB-231 cells (Fig. 6C). Collectively, these findings indicate that CAFs are likely a primary target through which H1R antagonists suppress EMT-related gene expression, likely via inverse agonist activity at the H1R.

### Cetirizine suppresses CAF-induced migration and EMT in 4T1 cells

**Fig. 7:**
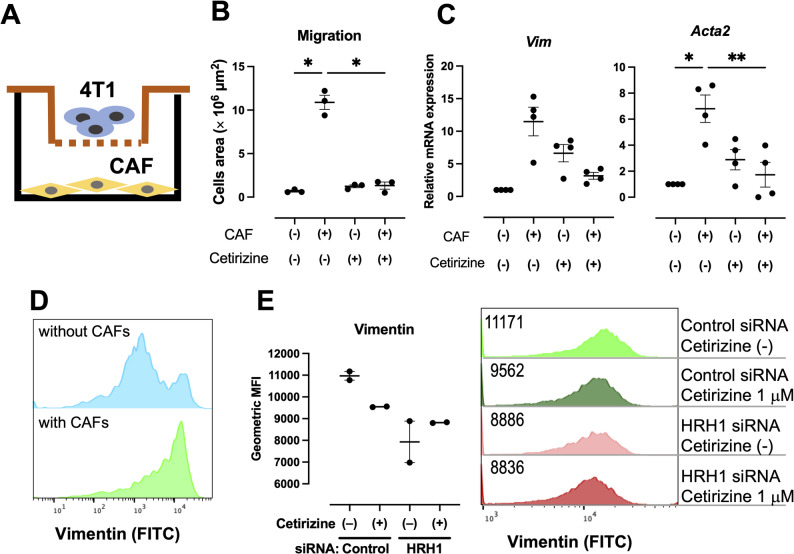
Cetirizine suppresses CAF-induced migration and EMT in 4T1 cells. **A** Schematic of the non-contact transwell co-culture system used to assess the effects of CAF-derived soluble factors on 4T1 cells. **B** Migration assay of 4T1 cells cultured alone or co-cultured with CAFs, in the absence or presence of cetirizine (1 µM). n = 3; data are shown as mean ± SEM. **C** Quantitative RT-PCR analysis of EMT markers (Vim, Acta2) in 4T1 cells under the same conditions as in (**B**). n = 4; data are shown as mean ± SEM. Each dot represents an individual sample. Statistical analysis was performed using repeated-measures one-way ANOVA followed by Bonferroni’s multiple comparison test. Exact P values are shown in the graphs. *P < 0.05, **P < 0.01. **D** Histograms showing vimentin expression in 4T1 cells cultured alone or in non-contact co-culture with CAFs. **E** (Left) Quantification of vimentin expression in 4T1 cells co-cultured with CAFs under the indicated conditions (control or HRH1 siRNA ± cetirizine [1 µM]). Data represent the mean of two independent experiments. (Right) Representative histograms of vimentin expression. Numbers in the plots indicate GeoMFI.

To investigate how cetirizine-induced changes in CAFs influence tumor cell migration and EMT, we used a non-contact transwell co-culture system (Fig. 7A). Although this system combines human-derived CAFs and mouse 4T1 cells, human-derived soluble factors such as TGF-β1 have been reported to induce EMT in 4T1 cells [13]. Compared to monoculture, co-culture with CAFs significantly enhanced the migratory capacity of 4T1 cells (Fig. 7B). This CAF-induced increase in migration was abolished in the presence of cetirizine. In contrast, in the absence of CAFs, cetirizine had no effect on migration. In parallel, co-culture with CAFs also increased the expression of EMT markers such as vimentin (Vim) and alpha-smooth muscle actin (Acta2) in 4T1 cells, and this effect was likewise suppressed by cetirizine (Fig. 7C). In the absence of CAFs, cetirizine had little or slightly increasing effect on EMT marker expression. At the protein level, flow cytometric analysis revealed that vimentin expression in monocultured 4T1 cells exhibited a bimodal distribution, whereas co-culture with CAFs shifted the population toward a predominantly high-expression peak (Fig. 7D). This CAF-induced increase in vimentin expression was suppressed by cetirizine. Furthermore, knockdown of HRH1 in CAFs attenuated the CAF-induced increase in vimentin expression in 4T1 cells, and cetirizine did not exert additional suppressive effects under HRH1-knockdown conditions (Fig. 7E). Collectively, these findings indicate that CAF-derived soluble factors promote mesenchymal and migratory changes in 4T1 cells in an H1R–dependent manner, and that cetirizine suppresses these processes likely by targeting H1R in CAFs.

## Discussion

This study identifies CAFs as a key target of the histamine H1R antagonist cetirizine and demonstrates that H1R–dependent regulation of CAFs is associated with modulation of EMT-related tumor plasticity in TNBC. These findings suggest that histamine signaling within the stromal compartment, rather than tumor cells themselves, plays an important role in regulating EMT-related programs in the tumor microenvironment. In this context, CAF-derived soluble factors are likely key mediators linking H1R activity to tumor cell plasticity, highlighting a stromal mechanism by which antihistamines can influence tumor progression.

A key finding of this study is that the effects of cetirizine on CAFs are mediated through the H1R at pharmacologically relevant concentrations. In contrast to previous studies in which antihistamines were used at relatively high micromolar concentrations and attributed EMT suppression to H1R–independent mechanisms [4, 21], the effects observed here occurred at nanomolar concentrations consistent with the known binding affinity of cetirizine. Moreover, cetirizine suppressed EMT-related gene expression in CAFs even under histamine-depleted conditions and failed to exert additional effects upon HRH1 knockdown, supporting that these effects depend on basal H1R activity. Together with the observed downregulation of HRH1 expression, these findings are consistent with inverse agonist activity of cetirizine in CAFs. These differences between the present findings and previous reports likely reflect variations in drug concentration, target cell type, and experimental context, suggesting that H1R–dependent and –independent effects are not mutually exclusive but operate under distinct conditions.

CAFs are major producers of ECM components and play a central role in tissue remodeling and fibrosis within the tumor microenvironment [3, 20]. In the present study, transcriptomic analysis of 4T1 tumors indicated that cetirizine primarily suppressed fibrosis- and extracellular matrix–related pathways, rather than EMT-specific programs, suggesting that its in vivo effects are dominated by modulation of fibrotic stromal processes. Consistently, cetirizine suppressed the expression of ECM-related genes and TGF-β1 in CAFs in vitro, indicating that H1R signaling contributes to the maintenance of a profibrotic microenvironment. Suppression of this axis by cetirizine was associated with reduced expression of fibrosis-related pathways in tumors, supporting attenuation of CAF activity. Given that ECM accumulation and TGF-β signaling are key drivers of both fibrosis and EMT, these findings suggest that cetirizine primarily acts by suppressing CAF-mediated fibrotic programs, which may in turn indirectly influence tumor cell plasticity. This mechanism is reminiscent of physiological fibroblast activation during wound healing [28], where H1R signaling promotes ECM production [18, 25], suggesting that analogous H1R–dependent processes may underlie both normal and pathological fibroblast activation.

The combination of cetirizine and aPD-1 resulted in enhanced tumor control compared with either monotherapy, accompanied by transcriptional changes indicative of increased immune activation. Notably, these changes were not associated with marked alterations in the abundance of major immune cell populations, suggesting that cetirizine may influence immune function rather than immune cell recruitment. Given the role of CAFs in shaping an immunosuppressive microenvironment through extracellular matrix deposition and cytokine production, attenuation of CAF activity by cetirizine may contribute to a more permissive environment for antitumor immunity. In this context, modulation of stromal components, rather than direct effects on immune cells, may represent an additional mechanism by which antihistamines enhance the efficacy of immune checkpoint blockade.

Cetirizine is a widely used second-generation antihistamine with a well-established safety profile that permits long-term administration [27]. The present findings suggest that cetirizine may be repurposed to target stromal components of the TME, particularly CAFs, to modulate EMT-related tumor plasticity. Given its clinical availability, this approach may offer a practical strategy to complement existing therapies, including immune checkpoint blockade, in TNBC. In addition, by analogy to the prophylactic use of H1R inverse agonists in nasal allergy [6], cetirizine may function as a pre-emptive modulator of a metastasis-permissive tumor microenvironment, acting prior to overt metastatic progression. However, the clinical relevance of HRH1 expression in relation to CAF abundance and patient outcomes in TNBC remains to be established. In addition, identification of predictive biomarkers—such as HRH1 expression, ECM-related signatures, or fibrotic gene scores—will be important to stratify patients who may benefit from cetirizine therapy.

## Conclusions

This study identifies H1R as a key regulator of CAF function in TNBC. Cetirizine suppressed CAF-mediated fibrotic programs and attenuated EMT-related tumor plasticity. These effects were observed at physiologically relevant concentrations and under histamine-depleted conditions, consistent with inverse agonist activity at H1R. Given its established safety profile and clinical availability, cetirizine represents a promising candidate for drug repurposing to modulate the tumor microenvironment, particularly stromal components, in TNBC.

## Supplementary Information

Below is the link to the electronic supplementary material.

Supplementary Material 1

Supplementary Material 2

Supplementary Material 3

Supplementary Material 4

Supplementary Material 5

Supplementary Material 6

## Data Availability

All data generated or analysed during this study are included in this published article and its supplementary information files.

## Abbreviations

αFMH: α-Fluoromethylhistidine
aPD-1: Anti-programmed cell death protein 1 antibody
ANOVA: Analysis of variance
BSA: Bovine serum albumin
CAF(s): Cancer-associated fibroblast(s)
ECM: Extracellular matrix
ELISA: Enzyme-linked immunosorbent assay
EMT: Epithelial–mesenchymal transition
ER: Estrogen receptor
FBS: Fetal bovine serum
GSEA: Gene set enrichment analysis
HBSS: Hanks’ balanced salt solution
HER2: Human epidermal growth factor receptor 2
H1R: Histamine H_1_ receptor
HUVEC(s): Human umbilical vein endothelial cell(s)
ICI(s): Immune checkpoint inhibitor(s)
IPA: Ingenuity pathway analysis
NES: Normalized enrichment score
PR: Progesterone receptor
RNA-seq: RNA sequencing
RT-PCR: Reverse transcription-polymerase chain reaction
SEM: Standard error of the mean
siRNA: Small interfering RNA
TCGA: The cancer genome atlas
TGF-β: Transforming growth factor-beta
TME: Tumor microenvironment
TNBC: Triple-negative breast cancer
