# Supplementary material for: Cetirizine suppresses cancer-associated fibroblast-mediated fibrotic remodeling and epithelial–mesenchymal transition via histamine H1 receptor inverse agonism
Source: Breast Cancer Res. 2026 Apr 26;28:113. doi: 10.1186/s13058-026-02284-x (PMC13285043; doi:10.1186/s13058-026-02284-x)
Supplement: Supplementary file 6 — Supplementary Material 6 [file 13058_2026_2284_MOESM6_ESM.docx]

**Supplementary material**

**Title**

Cetirizine suppresses cancer-associated fibroblast–mediated fibrotic remodeling and epithelial–mesenchymal transition via histamine H1 receptor inverse agonism

**Authors**

Aya Sasaki, Masahiro Hosonuma, Yuki Maruyama, Eiji Funayama, Hitoshi Toyoda, Toshiaki Tsurui, Kohei Tajima, Rie Nakashima, Yoshitaka Yamazaki, Akira Orimo, Tatsunori Oguchi, Kiyoshi Yoshimura, Atsuo Kuramasu*

***Corresponding author**

Atsuo Kuramasu

Department of Clinical Immuno Oncology, Clinical Research Institute for Clinical Pharmacology and Therapeutics, Showa Medical University, 6-11-11 Kita-Karasuyama, Setagaya-Ku, Tokyo, 157-8577, Japan.

atsuok@med.showa-u.ac.jp

**Contents:**

This file contains Supplementary Figures (S1–S4).

Supplementary Tables (S1–S4) are provided separately as an Excel file.

Supplementary Table S1: Gene set enrichment analysis results

Supplementary Table S2: RNA-seq expression data from 4T1 mouse tumors

Supplementary Table S3: Correlation analysis of gene expression, ρ vs P values

Supplementary Table S4: Ingenuity Pathway Analysis results of 4T1 tumors

Supplementary Table S5: Flow Cytometry Antibody Panels

**Supplementary Figure S1. Representative H&E images of spontaneous lung metastasis in the 4T1 subcutaneous tumor model.**
(A) Low-magnification image. The yellow square indicates the region shown at higher magnification in (B).
(B) High-magnification image of the boxed area in (A). Tumor cells are delineated by the yellow dashed line. Scale bar, 100 µm.

**Supplementary Figure S2. Gross images of lungs from the experimental lung metastasis model.**

**Supplementary Figure S3. Gating strategy for analysis of tumor cellular composition.**

Live singlets were identified by FSC-A/FSC-H gating and exclusion of FVS780⁺ dead cells. Cells were first separated on a CD31 vs CD45 plot to define immune cells (CD45⁺) and to gate the CD45⁻CD31⁻ population. The CD45⁻CD31⁻ fraction was subsequently analyzed for EpCAM and podoplanin expression to identify tumor-enriched (EpCAM⁺) and CAF-like (EpCAM⁻podoplanin⁺) cells. CD31 was used to exclude endothelial cells from the non-immune compartment.

**Supplementary Figure S4. Gating strategy for analysis of immune cells in tumor**(A) After exclusion of debris and doublets, live CD45⁺ cells were identified. Dendritic cells (DCs) were defined as CD11c⁺I-A/I-E⁺ within the CD45⁺ population. DCs were further subdivided based on XCR1 and CD11b expression to identify cDC1 (XCR1⁺) and cDC2 (CD11b⁺) subsets. The non-DC fraction (CD11c⁻ and/or I-A/I-E⁻) was subsequently analyzed using CD11b and F4/80 expression to identify macrophages (CD11b⁺F4/80⁺). (B) Lymphocytes were gated by FSC/SSC, followed by singlet discrimination and exclusion of dead cells using FVS780 to identify live CD45⁺ cells. CD8⁺ T cells were defined as CD3⁺CD8⁺, and effector-memory CD8⁺ T cells were identified as CD44⁺CD62L⁻. CXCR3 expression was analyzed by histogram.
